# Portrait of the Inflammatory Response to Radioiodine Therapy in Female Patients with Differentiated Thyroid Cancer with/without Type 2 Diabetes Mellitus

**DOI:** 10.3390/cancers15153793

**Published:** 2023-07-26

**Authors:** Adina Elena Stanciu, Anca Hurduc, Marcel Marian Stanciu, Mirela Gherghe, Dan Cristian Gheorghe, Virgiliu Mihail Prunoiu, Adina Zamfir-Chiru-Anton

**Affiliations:** 1 Department of Carcinogenesis and Molecular Biology, Institute of Oncology Bucharest, 022328 Bucharest, Romania; 2 Department of Radionuclide Therapy, Institute of Oncology Bucharest, 022328 Bucharest, Romania; ahurduc@yahoo.com; 3 Electrical Engineering Faculty, University Politehnica of Bucharest, 060042 Bucharest, Romania; marcel.stanciu@upb.ro; 4 Nuclear Medicine Department, Institute of Oncology Bucharest, 022328 Bucharest, Romania; 5 Nuclear Medicine Department, University of Medicine and Pharmacy “Carol Davila” Bucharest, 050474 Bucharest, Romania; 6 ENT Department, “Maria Sklodowska Curie” Children’s Emergency Hospital, 077120 Bucharest, Romania; gheorghe.dancristian@gmail.com; 7 ENT Department, University of Medicine and Pharmacy “Carol Davila” Bucharest, 050474 Bucharest, Romania; 8 Oncological Surgery Department, Institute of Oncology Bucharest, 022328 Bucharest, Romania; virgiliu.prunoiu@umfcd.ro; 9 Oncological Surgery Department, University of Medicine and Pharmacy “Carol Davila” Bucharest, 050474 Bucharest, Romania; 10 ENT Department, “Grigore Alexandrescu” Children’s Emergency Hospital, 011743 Bucharest, Romania; zamfiradina@yahoo.com

**Keywords:** differentiated thyroid cancer, type 2 diabetes mellitus, ^131^I, BMI, blood radioactivity, NLR, PLR

## Abstract

**Simple Summary:**

No clinical studies have investigated the effect of radioiodine (^131^I)-targeted therapy on the potential markers of ongoing inflammation in patients with DTC associated with T2DM and obesity. To our knowledge, this is the first study to report the relationship between prescribed ^131^I activity, blood radioactivity, BMI, and neutrophil-to-lymphocyte and platelet-to-lymphocyte ratios measured three days after ^131^I intake in DTC with/without T2DM patients. The strong correlation measured between blood radioactivity and high BMI, without a correlation between blood radioactivity and the ^131^I dose, proves a possible connection between ^131^I uptake in the bloodstream and chronic inflammation, the so-called “perfect storm”, specific to T2DM. Considering the immune response to ^131^I therapy, the two groups of patients can be seen as a synchronous portrait of two sides: in patients without T2DM, if ^131^I therapy has immunosuppressive effects, in the chronic inflammation context of T2DM, ^131^I therapy amplifies immune metabolism.

**Abstract:**

No clinical studies have investigated the effect of radioiodine (^131^I)-targeted therapy on the neutrophil-to-lymphocyte ratio (NLR) and platelet-to-lymphocyte ratio (PLR) as inflammatory response markers in patients with differentiated thyroid cancer (DTC) associated with type 2 diabetes mellitus (T2DM) and obesity. This study aimed to assess the relationship between blood radioactivity, body mass index (BMI), and peripheral blood cells three days after ^131^I intake in 56 female patients without T2DM (DTC/−T2DM) vs. 24 female patients with T2DM (DTC/+T2DM). Blood radioactivity, measured three days after ^131^I intake, was significantly lower in the DTC/+T2DM than in the DTC/−T2DM patients (0.7 mCi vs. 1.5 mCi, *p* < 0.001). The relationship between blood radioactivity and BMI (r = 0.83, *p* < 0.001), blood radioactivity and NLR (r = 0.53, *p* = 0.008), and BMI and NLR (r = 0.58, *p* = 0.003) indicates a possible connection between the bloodstream ^131^I uptake and T2DM-specific chronic inflammation. In patients without T2DM, ^131^I therapy has immunosuppressive effects, leading to increased NLR (19.6%, *p* = 0.009) and PLR (39.1%, *p* = 0.002). On the contrary, in the chronic inflammation context of T2DM, ^131^I therapy amplifies immune metabolism, leading to a drop in NLR (10%, *p* = 0.032) and PLR (13.4%, *p* = 0.021). Our results show that, in DTC/+T2DM, the bidirectional crosstalk between neutrophils and obesity may limit ^131^I uptake in the bloodstream. Considering the immune response to ^131^I therapy, the two groups of patients can be seen as a synchronous portrait of two sides. The explanation could lie in the different radiosensitivity of T and B lymphocytes, with T lymphocytes being predominant in patients with DTC/−T2DM and, most likely, B lymphocytes being predominant in T2DM.

## 1. Introduction

The International Diabetes Federation (IDF) Diabetes Atlas 10th edition estimates a continued global increase in the prevalence of diabetes mellitus (DM), from 537 million adults in 2021 to 643 million by 2030 and 783 million by 2045 [1]. The most frequent type of DM, type 2 diabetes (T2DM), is commonly associated with a high body mass index (BMI) (expressed by overweight and obesity) [2]. Previous studies and meta-analyses have provided substantial evidence of the associations between T2DM and an increased risk of liver, pancreas, gallbladder, colorectal, breast, thyroid, kidney, and bladder cancer [3]. Evidence suggests a strong underlying relationship between differentiated thyroid cancer (DTC), the most common endocrine malignancy, and T2DM [4]. Standard-of-care management for DTC includes risk-adapted surgery, postoperative radioiodine (^131^I) therapy based on the individual risk assessment, and thyroid hormone therapy [5,6,7,8,9]. Post-thyroidectomy suppression of thyroid-stimulating hormone (TSH) by high-dose levothyroxine can harm glucose homeostasis by impacting pancreatic β-cell development and glucose metabolism. A retrospective population-based cohort study revealed a U-shaped dose-dependent relationship between the levothyroxine dosage and T2DM [10].

The thyroid gland concentrates iodine via a sodium–iodide symporter (NIS) [11]. NIS has a longer half-life (5 days) in the presence of a high TSH level than in its absence (3 days) [11]. This explains why ^131^I requires a TSH level greater than 30 mIU/L to enhance iodine uptake by thyroid cancer cells. Moreover, the thyroid gland concentrates iodine by a factor of 20–40 times compared to plasma [12]. This characteristic is the basis for treating patients with thyroid cancers originating from cells that concentrate iodine. ^131^I-targeted therapy is based on delivering cytotoxic radiation levels to thyroid cancer cells or their microenvironment (presumably benign residual thyroid tissue, suspected but not identified remaining disease, and/or known residual or recurrent disease). However, some studies indicate the organification of iodine in tissues other than the thyroid, with NIS expression even being detected in the pancreas (ductal cells, exocrine parenchymal cells, and islets of Langerhans) [13,14]. Very few studies have analyzed the relationship between ^131^I and pancreatic function in DTC patients. The uptake of a large dose of ^131^I by the pancreas may affect β-cells, especially in patients diagnosed with T2DM [14]. Even if the ^131^I therapy has achieved its desired therapeutic success, there are still question marks related to cellular heterogeneity in the tumor mass and/or cellular radiobiological response in patients with DTC associated with T2DM (DTC/+T2DM). 

T2DM, historically considered a metabolic disease, is characterized by chronic systemic inflammation promoted by age and obesity. Inflammageing, the long-term result of the chronic physiological stimulation of the innate immune system, and metaflammation, a chronic low-grade inflammatory state orchestrated by metabolic cells in response to excess nutrients and energy (high BMI), represent the yin and yang of the T2DM [15,16]. Inflammation is widely acknowledged to play a significant role in the onset and progression of both cancer and T2DM and can be considered a hallmark of both diseases [3]. Chronic inflammation, the so-called “perfect storm”, is an extreme challenge that cancer and T2DM patients face. Growing evidence shows that chronic systemic inflammation and the patient’s immune status are closely related. Even though the overall mechanisms are not fully understood, radiation exposure is believed to have an ambivalent role on the immune system, exerting both immunogenic antitumor and immunosuppressive effects. Unlike conventional radiation therapy, targeted radionuclide therapy has a more pronounced effect on the immune system [14,17,18,19,20,21,22]. Recent data provide valuable information on potential markers of ongoing inflammation, including neutrophil counts, platelet counts, lymphocyte counts, platelet-to-lymphocyte ratio (PLR), and neutrophil-to-lymphocyte ratio (NLR) [18,19,20,21,22] after ^131^I therapy. 

While the number of publications investigating the effect of targeted therapy with ^131^I on these potential new indicators of chronic inflammation in patients with DTC is relatively limited [18,19,20,21,22], they are practically nonexistent in patients with DTC associated with T2DM. This study aims to strengthen the results obtained by us previously [21,23]. Based on our results showing a higher incidence of platelets and lymphocytes in DTC/+T2DM patients than in DTC/−T2DM [23], we hypothesized that the effect of ^131^I therapy on the peripheral blood cells measured in the two groups should also differ. To our knowledge, this is the first study to report the relationship between the prescribed ^131^I activity, whole-blood radioactivity, BMI, NLR, and PLR measured three days after ^131^I intake in patients with DTC/+T2DM vs. DTC/−T2DM. 

## 2. Materials and Methods

### 2.1. Patients and Study Protocol

This prospective study included 56 female patients with DTC/−T2DM (mean age, 57.3 ± 9.1 years) and 24 female patients with DTC/+T2DM (mean age, 61.6 ± 6.9 years) who were referred to the Department of Radionuclide Therapy of the Institute of Oncology Bucharest for targeted therapy with ^131^I over a period of approximately two years between 2019 and 2020. The enrolled patients discontinued L-thyroxine treatment and followed a low-iodine diet for at least four weeks and two weeks, respectively, before the administration of ^131^I sodium iodide ThyroTop, a radiopharmaceutical purchased from the Institute of Isotopes Co. Ltd. (Budapest, Hungary). For a strong uptake into the thyroid bed, ^131^I sodium iodide ThyroTop was administered when the TSH level was higher than 30 IU/mL after fasting for more than 4 h and continuing to fast for another 2 h. The ^131^I given activity in this patient cohort was based on the joint statement on the theranostic approaches in the management and therapy of DTC [5,6] by the European Thyroid Association (ETA), American Thyroid Association (ATA), Society of Nuclear Medicine and Molecular Imaging (SNMMI), and the European Association of Nuclear Medicine (EANM), as well as the recent SNMMI/EANM practice guideline [8] and ETA Consensus [9], in accordance with safety precautions [24]. Patients were instructed to maintain adequate hydration after receiving the therapeutic dose of ^131^I to enable frequent urination and quickly eliminate the ^131^I from the body.

The following were the exclusion criteria: (i) age under 18 years; (ii) acute or chronic infection (known to affect the NLR and PLR value); (iii) renal impairment (kidney function is involved in ^131^I elimination) [21]; (iv) gastrointestinal disease (stool frequency); (v) ongoing diuretic treatment (urine frequency); (vii) ongoing treatment with steroidal or non-steroidal anti-inflammatory drugs (known to affect the NLR and PLR value) [25]; (vi) ongoing treatment with aspirin (known to affect the platelets and PLR value); (vii) a history of coronary heart disease, cerebral infarction, and blood system disease(s) that would possibly affect platelet- and PLR-related indices; (viii) poorly controlled diabetes (known to lead to insulin resistance and hyperinsulinemia, which causes thyroid tissue proliferation) [4]. Because antidiabetics such as sulfonylureas, pioglitazone, and thiazolidinediones can have a negative impact on thyroid disease [4], the DTC/+T2DM patients were treated with metformin, which is beneficial for both T2DM and DTC. To avoid intersex variations, only women were included in the study. 

The medical and medication histories and demographic data (age, height, weight) of each enrolled patient were compiled. The serum TSH level measured upon admission to the Department of Radionuclide Therapy was collected from medical records. The blood volume and body mass index (BMI) of the recruited individuals were computed using their height (H) and weight (W). The Nadler equation for females was used to obtain the whole blood volume/patient: ${Blood}_{Volume}=\left(0.3561xH^{3}\right)+\left(0.03308xW\right)+0.1833$. The patient’s kilograms (kg) weight was divided by their height in meters squared (m^2^) to calculate BMI. BMIs between 18.5 and 24.9 kg/m^2^ are considered to be optimum. Overweight is defined as a BMI between 25 and 29.9 kg/m^2^. In comparison, obesity is defined as a BMI equal to or greater than 30 kg/m^2^ (obesity class I: BMI = 30 to 34.9 kg/m^2^, obesity class II: BMI = 35 to 39.9 kg/m^2^, and obesity class III: BMI greater than or equal to 40 kg/m^2^) [26].

The study was conducted following the guiding principles of the Declaration of Helsinki and was approved by the local ethics committee of our institution (No.15140/10.09.2019). The research objectives and the medical procedure required for blood sampling were explained to each subject. At admission, each participant signed an informed consent form.

### 2.2. Blood Sampling and Radioactivity Quantification

Peripheral blood samples were extracted through intravenous puncture into BD Vacutainer EDTA tubes (purchased from Becton, Dickinson, and Company, Franklin Lakes, NJ, USA) with a draw volume of 2 mL before and three days after the therapeutic dose of ^131^I administration (immediately before the patient’s discharge from the isolation ward). As the blood samples collected after the ^131^I intake were still radioactive, each tube was tagged with an international radioactivity symbol sticker. The radioactivity of each blood sample tube (2 mL) was measured three times in a dose calibrator (CURIEMENTOR^R^ 4 Isotope Calibrator, PTW Freiburg, Freiburg, Germany). The background count was assessed before each radioactivity measurement and then subtracted from the registered value. The measurements ranged from 0.01 µCi to 2486 mCi (or from 0.001 mBq to 92 GBq), with an accuracy of about 5%. The blood radioactivity per 1 mL was then calculated by dividing the average of the three radioactivity measurements per blood sample tube (2 mL) by 2. Then, the blood sample radioactivity per 1 mL was multiplied by the patient’s blood volume to obtain each patient’s whole blood radioactivity.

A procedure has been developed for collecting, transporting, processing, and disposing of radioactive blood tubes. This procedure adheres to the current recommendations issued by the European Association of Nuclear Medicine (EANM) Dosimetry Committee [27].

### 2.3. Biomarker Measurement

Blood cell analysis (blood-count parameters: neutrophils, platelets, and lymphocytes) was conducted using an ADVIA 2120i automatic Hematology System with an auto slide (Siemens, Munich, Germany). The PLR and NLR were computed by dividing the absolute platelet count by the absolute lymphocyte count and the absolute neutrophil count by the absolute lymphocyte count, respectively. 

### 2.4. Statistics

Patient data were processed using Microsoft Office Excel 2007 SP2 (including Data Analysis Tools). Statistica software (version 8.0; StatSoft, Inc., Tulsa, OK, USA) was used for statistical analysis. Continuous variables were expressed as the means ± standard deviation or median value with interquartile range (IQR: 25–75%) when appropriate. The data acquired after preliminary analysis were verified using the Anderson–Darling, Shapiro–Wilk, and Kolmogorov–Smirnov tests. The non-parametric Kruskal–Wallis test was used to compare the distribution of continuous variables between different categories for independent samples (DTC/−T2DM vs. DTC/+T2DM groups), while the Wilcoxon test was used for paired samples (DTC/−T2DM group before and three days after ^131^I intake and DTC/+T2DM before and three days after ^131^I intake). The correlation between circulating biomarkers and their ratios was assessed using Pearson’s correlation coefficient (r). For all tests, significance was set at a *p*-value < 0.05.

## 3. Results

### 3.1. Blood Parameters before the ^131^I Intake

Several blood parameters measured in the two study groups before the ^131^I sodium iodide ThyroTop intake are summarized in Table 1. Significant differences in absolute lymphocyte and platelet counts were observed among the DTC/−T2DM and DTC/+T2DM patients (1.6 × 10^9^/L vs. 1.9 × 10^9^/L, *p* = 0.015 and 233.5 × 10^9^/L vs. 333 × 10^9^/L, *p* < 0.001). Increased platelet and lymphocyte counts were reported in female patients with concomitant T2DM. The median TSH level, confirming the short hypothyroid state specific to the four weeks of thyroid hormone withdrawal, was comparable (81.2 mIU/L in DTC/−T2DM patients vs. 81.3 mIU/L in DTC/+T2DM patients), with no statistical significance between the two groups.

### 3.2. Characteristics of the Study Population with a Focus on Whole-Blood Radioactivity

There were no significant differences between the two groups (DTC/−T2DM and DTC/+T2DM) regarding age (*p* = 0.153). BMI analysis showed that, unlike the DTC/−T2DM patients who were overweight, DTC/+T2DM patients suffered from obesity (obesity class I), with a median BMI of 33.3 kg/m^2^ (*p* < 0.001) (Table 2). Based on height and weight, the median volume of blood per patient was higher in the subjects with concomitant T2DM than in those without (4880.1 mL vs. 4363.1 mL, *p* = 0.018). Regarding whole-blood radioactivity, even though the administered median activity of ^131^I was higher in the DTC/+T2DM group than in DTC/−T2DM (107.9 mCi vs. 88.7 mCi or 3.992 GBq vs. 3.282 GBq, *p* = 0.035), the blood radioactivity measured three days after ^131^I intake was significantly lower in the DTC/+T2DM patients than in those with DTC/−T2DM (0.7 mCi vs. 1.5 mCi, *p* < 0.001).

### 3.3. Dynamics of Hematological Parameters after the ^131^I Intake

**Figure 1:**
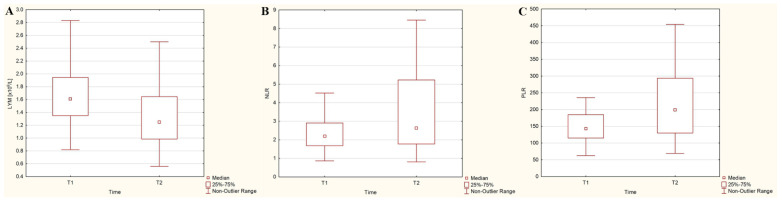
Dynamics of absolute lymphocyte counts (LYM) (**A**), neutrophil-to-lymphocyte ratio (NLR) (**B**), and platelet-to-lymphocyte ratio (PLR) (**C**) three days after ^131^I administration in differentiated thyroid cancer patients without type 2 diabetes mellitus.

Figure 1 illustrates the dynamics of absolute lymphocyte counts and the ratios (NLR, PLR) after ^131^I intake in the DTC/−T2DM group of patients. Lymphocyte count exhibited a decreasing trend of 20% (*p* = 0.015), while neutrophil count, platelet count, and NLR and PLR displayed an opposite trend, with increases of 9%, 11.1%, 19.6%, and 39.1%, respectively (*p* = 0.532, *p* = 0.034, *p* = 0.009 and *p* = 0.002, respectively), in the DTC/−T2DM patients following the administration of the recommended dose of ^131^I. 

**Figure 2:**
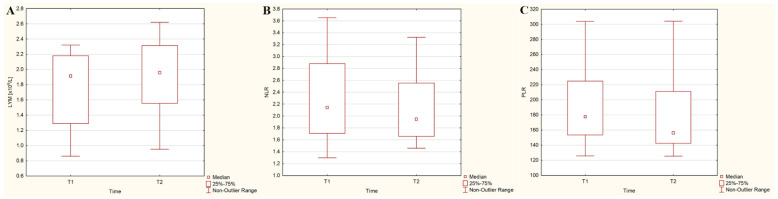
Dynamics of absolute lymphocyte counts (LYM) (**A**), neutrophil-to-lymphocyte ratio (NLR) (**B**), and platelet-to-lymphocyte ratio (PLR) (**C**) three days after ^131^I administration in differentiated thyroid cancer patients with type 2 diabetes mellitus.

In contrast, in the DTC/+T2DM group, the time course of lymphocytes, neutrophils, and platelets did not show statistically significant changes (8% increase—Figure 2A, 1.7% increase, and 1% decrease, respectively; *p* > 0.05). However, NLR and PLR exhibited a statistically significant decrease of 10% (*p* = 0.032) and 13.4% (*p* = 0.021) three days after ^131^I administration, as depicted in Figure 2B,C. 

### 3.4. Correlations in the DTC/−T2DM Group 

**Figure 3:**
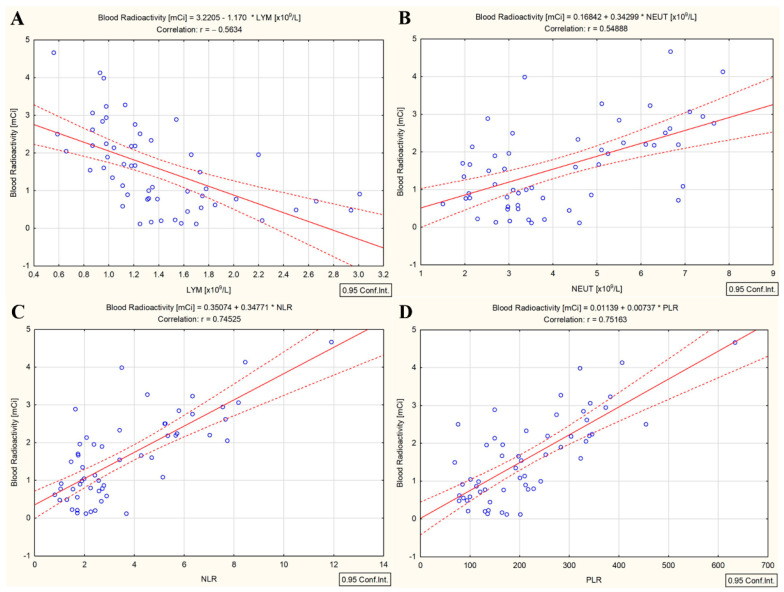
Correlation between the whole-blood radioactivity and absolute lymphocyte count (**A**), absolute neutrophile count (**B**), NLR (**C**), and PLR (**D**) in differentiated thyroid cancer patients without type 2 diabetes mellitus (measured three days after ^131^I intake); (“—” fitted linear regression curve, “- - -” equality line).

In the DTC/−T2DM group, there was a positive relationship between the administered ^131^I dose and whole-blood radioactivity measured three days after ^131^I intake (r = 0.64, *p* < 0.001), as we previously showed [21]. It should be mentioned that the BMI of the patients enrolled in this group was not correlated with whole-blood radioactivity. Furthermore, both administered ^131^I activity and whole-blood radioactivity were correlated with absolute lymphocyte count (r = −0.51 and r = −0.56, *p* < 0.001), absolute neutrophil count (r = 0.49 and r = 0.54, *p* < 0.001), and absolute platelet count (r = 0.46 and r = 0.51, *p* < 0.001) and strongly correlated with NLR (r = 0.69 and r = 0.74, *p* < 0.001) and PLR (r = 0.69 and r = 0.75, *p* < 0.001). The relationship between whole-blood radioactivity and absolute lymphocyte count, absolute neutrophile count, NLR, and PLR is presented in Figure 3. 

### 3.5. Correlations in the DTC/+T2DM Group

**Figure 4:**
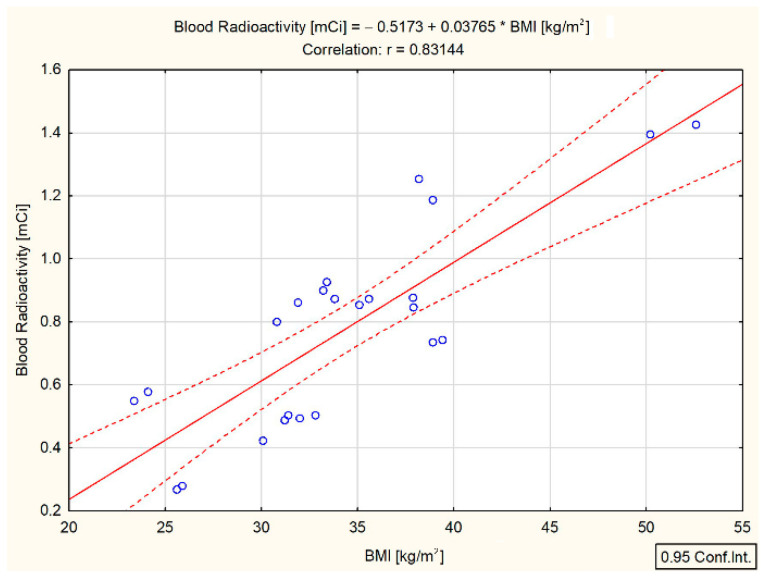
Correlation between whole-blood radioactivity and BMI in differentiated thyroid cancer patients with type 2 diabetes mellitus (measured at three days after ^131^I intake); (— fitted linear regression curve, - - - equality line).

Unlike the DTC/−T2DM group, the presence of T2DM led to a different kind of portrait of the relationships between the investigated parameters. An overview of the results presented in Figure 4 shows that whole-blood radioactivity was positively correlated with BMI (r = 0.83, *p* < 0.001). However, unexpectedly, whole-blood radioactivity was not correlated with the administered dose of ^131^I. 

**Figure 5:**
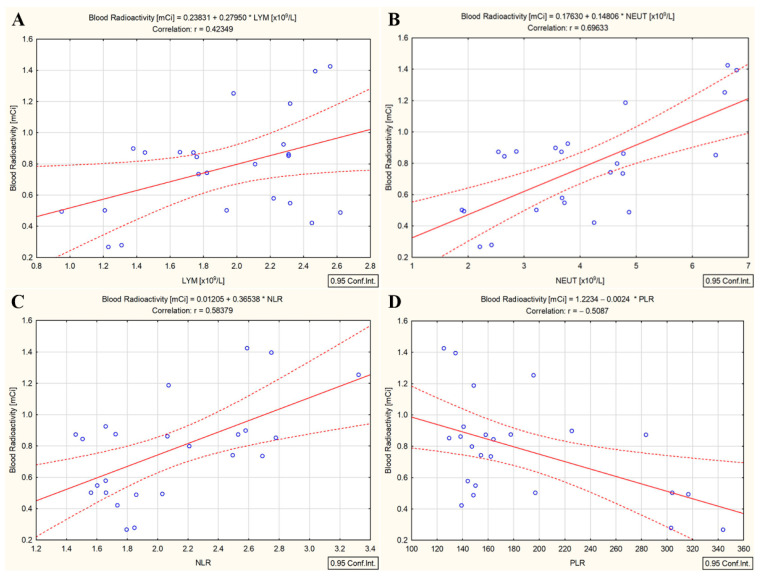
Correlation between whole-blood radioactivity and absolute lymphocyte count (**A**), absolute neutrophile count (**B**), NLR (**C**), and PLR (**D**) in differentiated thyroid cancer patients with type 2 diabetes mellitus (measured three days after ^131^I intake); (“—” fitted linear regression curve, “- - -” equality line).

Likewise, the ^131^I activity administered to the patients was not correlated with any investigated hematological parameter. A significant statistical relationship was measured only between whole-blood radioactivity and absolute lymphocyte count (r = 0.42, *p* = 0.039) (Figure 5A), absolute neutrophil count (r = 0.69, *p* < 0.001) (Figure 5B), NLR (r = 0.58, *p* = 0.003) (Figure 5C), and PLR (r = −0.50, *p* = 0.11) (Figure 5D).

**Figure 6:**
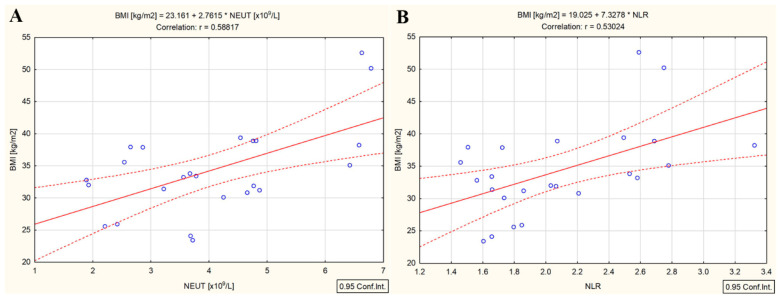
Correlation between the BMI and absolute neutrophile count (**A**) and NLR (**B**) in differentiated thyroid cancer patients with type 2 diabetes mellitus (measured three days after ^131^I intake); (“—” fitted linear regression curve, “- - -” equality line).

BMI was only correlated with neutrophils and NLR both before (r = 0.57, *p* = 0.004 and r = 0.54, *p* = 0.008, respectively) and after ^131^I administration (r = 0.58, *p* = 0.003 and r = 0.53, *p* = 0.008, respectively) (Figure 6A,B). 

## 4. Discussion

The present study reveals several key findings: Firstly, the blood radioactivity measured three days after ^131^I intake was significantly lower in the DTC/+T2DM patients compared to the DTC/−T2DM patients, despite higher administered doses and BMI values. Secondly, there was a strong positive correlation between blood radioactivity and BMI (r = 0.83, *p* < 0.001) in the DTC/+T2DM patients, but no correlation was observed with the administered dose of ^131^I. Thirdly, ^131^I therapy demonstrated immune-suppressive effects, resulting in increased NLR and PLR in the DTC/−T2DM patients. Lastly, in the context of chronic inflammation in T2DM, ^131^I therapy amplified immune metabolism, leading to a decrease in NLR and PLR.

Our study found that the whole-blood radioactivity measured three days after the ^131^I intake was significantly lower in the DTC/+T2DM patients than in the DTC/−T2DM patients (*p* < 0.001), despite higher administered doses and BMI (*p* = 0.035 and *p* = 0.012). These results align with our previous publication [21], which demonstrated a biphasic course of ^131^I blood concentration in DTC/−T2DM patients. The initial rapid decrease in whole-blood radioactivity was attributed to fast clearance by the kidneys, followed by a slight increase due to the release of ^131^I in the blood by residual thyroid tissue. Further investigations are needed to determine if DTC/+T2DM patients exhibit a similar biphasic course of ^131^I blood concentration. Nevertheless, our results confirm the theory proposed in other studies that the iodine uptake in the blood of T2DM patients is lower compared to healthy individuals. Several explanations could account for this difference:

(i) Expression of NIS: NIS expression has been detected in various tissues, including the pancreas, where ductal cells, exocrine parenchymal cells, and islets of Langerhans show positive staining [13,14]). It is possible that, in the presence of T2DM, iodine uptake is relatively high in pancreatic tissues, especially in the islets of Langerhans, which are known to exhibit dysfunction in T2DM [10,14]. It is well known that the thyroid gland concentrates iodine by a factor of 20–40 times compared to plasma [12]. Cumulatively, these factors may contribute to decreased ^131^I uptake in the bloodstream.
(ii) Changes in biomolecule conformation: The uptake of ^131^I in the blood is dependent on the iodination of proteins, carbohydrates, and lipids [28]. In the presence of T2DM, structural conformational changes in biomolecules from the blood, resulting from the disease itself, could reduce the number of binding sites and consequently decrease ^131^I uptake in this group [28].
(iii) Increased urination: Obesity, often associated with T2DM, can lead to increased intra-abdominal pressure, resulting in increased urine production or frequency [29]. This, coupled with the common symptom of increased urination in T2DM [30], could lead to a higher excretion of ^131^I in T2DM patients compared to those without T2DM.

The correlation between BMI and the administered radiation dose has been extensively studied in various settings, starting from diagnosis (mammography or computed tomography) to conventional radiotherapy. However, studies examining the correlation between BMI, administered dose, and external dose rates specifically for patients treated with ^131^I have produced contradictory results. Our findings are consistent with those obtained by Lahfi et al. [31] and Pickering et al. [32], who reported higher external dose rate values, with 11% and 10%, respectively, in the normal BMI group compared to the obese group for administered ^131^I doses exceeding 150 mCi. Lahfi et al. [31] attributed these results to increased gamma ray attenuation in obese patients. However, this explanation does not apply when analyzing whole-blood radioactivity. In our study, whole-blood radioactivity was 53% higher in the group without T2DM than in the obese group with T2DM. Notably, a statistically significant correlation between whole-blood radioactivity and BMI was found only in the presence of T2DM (r = 0.83, *p* < 0.001), as shown in Figure 4. Moreover, contrary to expectations, in the DTC/+T2DM patients, whole-blood radioactivity was not correlated with the administrated dose of ^131^I. 

The strong correlation measured between the low whole-blood radioactivity and high BMI (Figure 4) implies the existence of a possible connection between the ^131^I uptake in the bloodstream and the specific systemic chronic inflammation of T2DM (chronic low-grade inflammation of the adipose tissue along with chronic physiological stimulation of the innate immune system). One of the concepts explaining the association between obesity and T2DM in our study, obesity class I (BMI = 33.3 kg/m^2^) in DTC/+T2DM patients, is the theory of chronic, low-grade systemic inflammation orchestrated by the metabolic cells in response to excess nutrients and energy, called metaflammation. As additional evidence of this connection between the ^131^I uptake in the bloodstream and chronic low-grade inflammation of the adipose tissue, both BMI and whole-blood radioactivity were positively correlated with absolute neutrophil count (r = 0.58, *p* = 0.003 and r = 0.69, *p* < 0.001) (Figure 6A and Figure 5B) and NLR (r = 0.53, *p* = 0.008 and r = 0.58, *p* = 0.003) (Figure 6B and Figure 5C). It seems that the administration of the therapeutic dose of ^131^I led to the activation of immune metabolism. Immune metabolism refers to the interaction between metabolism and immune response to the action of targeted radionuclide therapy. It is well known that, during obesity-induced chronic inflammation, the number of neutrophils increases in the first phase in the peripheral circulation, and from there, neutrophils begin to infiltrate the adipose tissue [33] and the endothelium of blood vessels [34]. Consequently, in obesity-induced chronic inflammation, neutrophils are the first immune cells recruited in adipose tissue [35], and NLR is the expression of the relationship between innate (neutrophils) and adaptive (lymphocytes) immune responses. It seems that, in the presence of T2DM, the bidirectional crosstalk between neutrophils and obesity (statistically significant correlation between high BMI and neutrophils) leads to the limitation of ^131^I uptake in the bloodstream (statistically significant correlation between high BMI, neutrophils, and whole-blood radioactivity). 

The most severe side effect of radiotherapy in cancer patients is the suppression of hematopoiesis. Its curative effect is mainly based on modulating the immune response in the chronic inflammatory tissue. Therefore, systemic inflammation and their immune status are essential parameters influencing the response to radiotherapy. Although the overall mechanisms are not entirely understood, targeted radionuclide therapy (in our study, therapy with high doses of ^131^I) exerts both anti-tumor immunogenic and immune-suppressive effects. Practically, radionuclide therapy assumes that cells are not only irradiated for minutes as in conventional radiotherapy but are continuously irradiated over a much more extended period (with the intensity of the dose decreasing over time) depending on the effective half-life of the radionuclide. This continuous exposure of the peripheral blood cells to ^131^I with a median whole-body effective half-life of 13 h results in cell renewal, apoptosis, and the redistribution of hematopoietic cells, with hematological toxicity being the most common adverse effect of ^131^I therapy [18,22]. In our study, ^131^I intake led to a 22.4% drop in lymphocyte count and an increase of 15.4% in NLR and 28.1% in PLR in the DTC/−T2DM patients (Figure 1). These results align with studies by Yi et al. [22] and Rui et al. [18], which reported a 37% and 11.5% reduction in lymphocyte count following^131^I therapy. Because peripheral blood lymphocytes are the most radiosensitive mammalian cells, they are constantly regenerating, and their drop after irradiation confirms this. Moreover, the whole-blood radioactivity measured three days after the ^131^I intake was negatively correlated with the absolute lymphocyte count (r = −0.56, *p* < 0.001), as shown in Figure 3A. If, in the DTC/−T2DM group, the lymphocyte count decreased, in the presence of T2DM, the count increased by 2.6%; however, this increase was without statistical significance. This slight increase was marked by the positive correlation between the whole-blood radioactivity measured three days after ^131^I intake and the absolute lymphocyte count at this time (Figure 5A). An explanation for this could lie in the different radiosensitivity of T and B lymphocytes [36], T lymphocytes predominating in patients with DTC/−T2DM and, most likely, B lymphocytes in T2DM. Even if macrophages are the most abundant type of immune cell in adipose tissue, other immune cells, such as B cells, are also present and play an essential role in regulating adipose tissue inflammation in T2DM, with even a higher ratio of B-cell subsets than bone marrow [37].

Because lymphocytes are a mainstay of anticancer immunity, NLR and PLR reflect immune status after radiotherapy. The overall picture in which targeted radionuclide therapy exerts immune-suppressive effects leading to the increase in NLR and PLR in DTC/−T2DM patients was also shown by Chew et al. [17] after ^90^Y radioembolization in hepatocellular carcinoma. In our study, whole-blood radioactivity was strongly positively correlated with NLR (r = 0.74, *p* < 0.001) and PLR (r = 0.75, *p* < 0.001) in the DTC/−T2DM group (Figure 3C,D). In these patients, the local and systemic immune systems were activated differently by ^131^I therapy than by conventional radiotherapy, leading to sustained tumor response. If conventional radiotherapy induces apoptosis, targeted radionuclide therapy can predominantly induce necrosis, which is considered inflammatory cell death [38]. Therefore, the increased production of cytokines and chemokines leads to the activation of immune cells. Further, the enhanced immune system leads to increased NLR and PLR, demonstrating the immune-modulatory effects of ^131^I in a clinical setting. This picture is changed in the presence of T2DM because, after ^131^I irradiation, NLR dropped by 10%, and PLR dropped by 13.4%. Moreover, whole-blood radioactivity measured three days after the ^131^I intake was positively correlated with NLR (r = 0.58, *p* = 0.003) (Figure 5C) and negatively correlated with PLR (r = −0.50, *p* = 0.11) (Figure 5D). Because our patients did not receive any additional treatment apart from ^131^I, the changes in NLR and PLR are considered to be solely attributed to ^131^I. These observations suggest that the immunomodulatory effects of ^131^I therapy differ between patients with and without T2DM.

The limitations of our study include its single-center cross-sectional design and relatively small sample size. Despite these limitations, the study included patients referred from across the country to our Department of Radionuclide Therapy. Additionally, the sample size, although small, was sufficient for statistical calculations with a 95% confidence interval and a Z-score of 1.96. Furthermore, the sample size reflects the rarity of the association between DTC and T2DM. On the other hand, all blood samples collected three days after ^131^I intake were radioactive. The risk of healthcare professionals’ radioactive contamination must be considered. Moreover, the study population consisted solely of women due to sex-related differences in clinical outcomes and result interpretation, as sex hormones significantly influence the regulation of thyroid and immune functions. Between estradiol, progesterone, androgens, thyroid hormones, and the immune system is a complex crosstalk [39]. Sex hormones can modulate the immune response to ^131^I therapy. If, in women, progesterone has extensive anti-inflammatory effects and estradiol improves cellular and humoral mediated immune responses, in men, androgens (dihydrotestosterone and testosterone) suppress the activity of immune cells, leading to low immune responses [40]. The immune system functions in a sexually dimorphic manner, with females exhibiting more robust immune responses than males [41].

## 5. Conclusions

In conclusion, our study reveals correlations between whole-blood radioactivity, BMI, and NLR, indicating a possible connection between ^131^I uptake in the bloodstream and the chronic inflammation characteristic of T2DM. The bidirectional crosstalk between neutrophils and obesity appears to limit ^131^I uptake in the bloodstream in the presence of T2DM. Moreover, ^131^I therapy demonstrated immunosuppressive effects in the DTC/−T2DM patients, leading to increased NLR and PLR. In contrast, in the context of chronic inflammation in T2DM, ^131^I therapy amplified immune metabolism, resulting in decreased NLR and PLR. These findings suggest that reducing B lymphocytes could be a potential treatment direction for DTC/+T2DM patients. Further research is needed to elucidate the underlying mechanisms and explore the relationship between prescribed ^131^I activity, whole-blood radioactivity, and peripheral blood cells at multiple time points in DTC/+T2DM patients.

## Figures and Tables

**Table 1:** Blood parameters measured before ^131^I intake in the two study groups.

Variables	DTC/−T2DM	DTC/+T2DM	*p*-Value
	*n* = 56	*n* = 24	
Lymphocytes (×10^9^/L) ^a^	1.6 (1.3–1.9)	1.9 (1.3–2.2)	0.015
Neutrophils (×10^9^/L) ^a^	3.6 (3.1–4.5)	3.7 (3.0–4.6)	0.731
Platelets (×10^9^/L) ^a^	233.5 (199.0–273.0)	333.0 (312.0–378.0)	<0.001
NLR ^a^	2.2 (1.7–2.9)	2.1 (1.7–2.8)	0.712
PLR ^a^	143.1 (115.1–184.5)	177.5 (153.5–224.8)	0.035
TSH (mIU/L) ^a^	81.2 (62.7–98.5)	81.3 (62.9–99.1)	0.752

DTC/−T2DM, differentiated thyroid cancer without type 2 diabetes mellitus; DTC/+T2DM, differentiated thyroid cancer with type 2 diabetes mellitus; NLR, neutrophil-to-lymphocyte ratio; PLR, platelet-to-lymphocyte ratio; ^131^I, radioiodine; TSH, thyroid stimulating hormone; ^a^ Data are expressed as median and interquartile ranges (25–75%).

**Table 2:** Clinical data in the two study groups.

Variables	DTC/−T2DM	DTC/+T2DM	*p*-Value
	*n* = 56	*n* = 24	
Age (years) ^a^	57.3 ± 9.1	62.7 ± 6.5	0.153
Height (m) ^b^	1.64 (1.60–1.65)	1.63 (1.60–1.65)	0.754
Weight (kg) ^b^	81.8 (69.05–90.74)	91.48 (80.12–101.44)	0.032
BMI (kg/m^2^) ^b^	29.6 (26.2–33.9)	33.3 (30.8–37.9)	<0.001
Blood Volume (mL) ^b^	4364.1 (3976.3–4825.1)	4880.1 (4613.8–5112.3)	0.018
Administered Activity of ^131^I (mCi) ^b^	88.7 (60.7–129.4)	107.9 (88.9–137)	0.035
Whole-blood radioactivity (mCi) ^b^	1.5 (0.7–2.3)	0.7 (0.4–0.9)	<0.001

BMI, body mass index; DTC/−T2DM, differentiated thyroid cancer without type 2 diabetes mellitus; DTC/+T2DM, differentiated thyroid cancer associated with type 2 diabetes mellitus; ^131^I, radioiodine; ^a^ mean ± standard deviation; ^b^ Data are expressed as median and interquartile ranges (25–75%).

## Data Availability

The data presented in this study are available upon request from the corresponding author.
